# Supplementary material for: Synthesis and Characterization of Calcium Silicate Nanoparticles Stabilized with Amino Acids
Source: Micromachines (Basel). 2023 Jan 18;14(2):245. doi: 10.3390/mi14020245 (PMC9967975; doi:10.3390/mi14020245)
Supplement: Supplementary file 1 [file micromachines-14-00245-s001.zip › micromachines-2103473-supplementary.pdf]

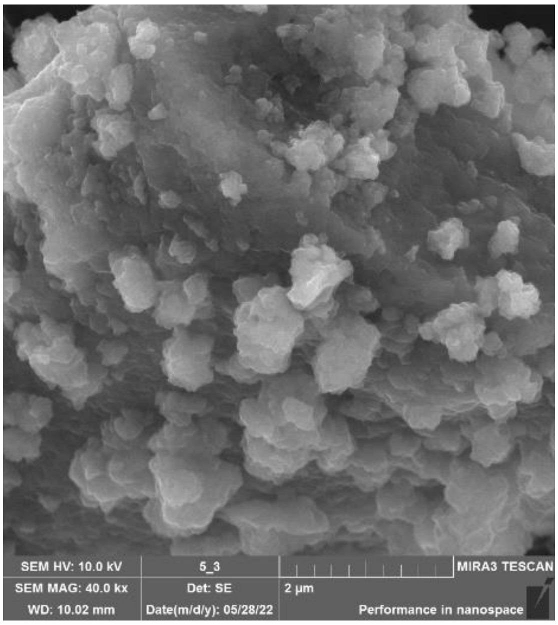
SEM HV: 10.0 kV
5_3
MIRA3 TESCAN
SEM MAG: 40.0 kx
Det: SE
2 µm
WD: 10.02 mm
Date(m/d/y): 05/28/22
Performance in nanospace

a

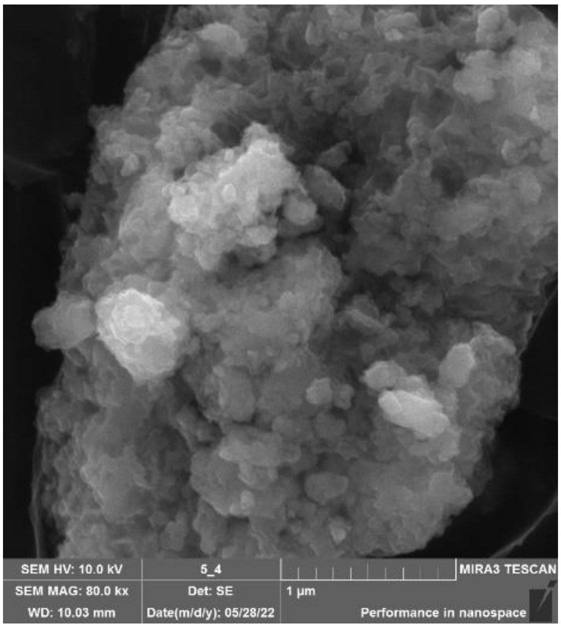
SEM HV: 10.0 kV
5_4
MIRA3 TESCAN
SEM MAG: 80.0 kx
Det: SE
1 µm
WD: 10.03 mm
Date(m/d/y): 05/28/22
Performance in nanospace

b

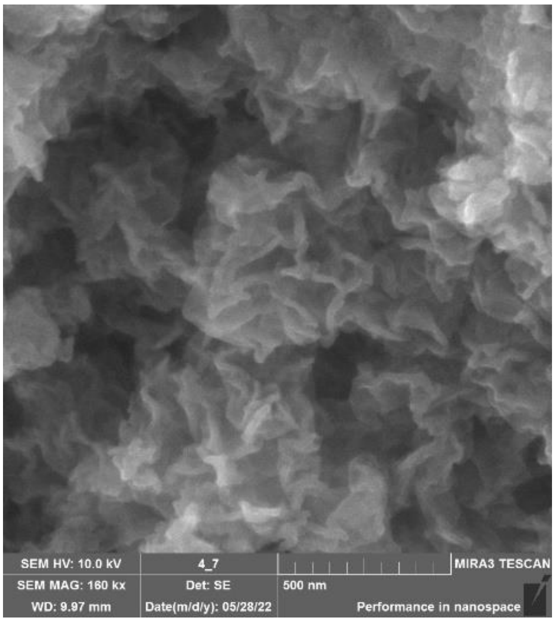
SEM HV: 10.0 kV
4_7
MIRA3 TESCAN
SEM MAG: 160 kx
Det: SE
500 nm
WD: 9.97 mm
Date(m/d/y): 05/28/22
Performance in nanospace

c

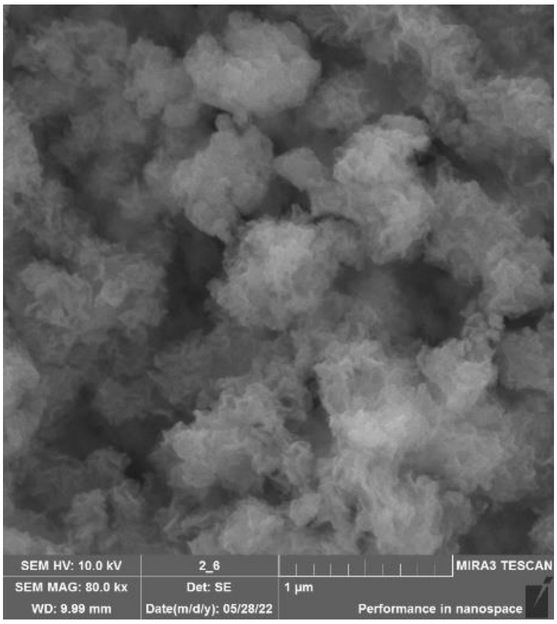
SEM HV: 10.0 kV
2_6
MIRA3 TESCAN
SEM MAG: 80.0 kx
Det: SE
1 µm
WD: 9.99 mm
Date(m/d/y): 05/28/22
Performance in nanospace

d

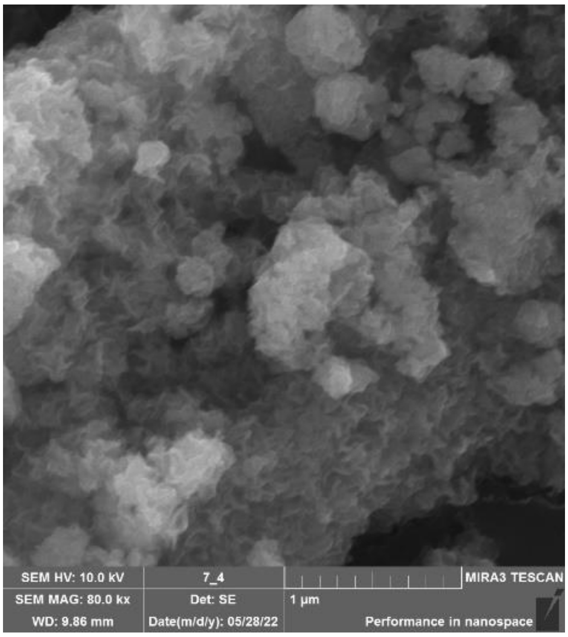
SEM HV: 10.0 kV
7_4
MIRA3 TESCAN
SEM MAG: 80.0 kx
Det: SE
1 µm
WD: 9.86 mm
Date(m/d/y): 05/28/22
Performance in nanospace

e

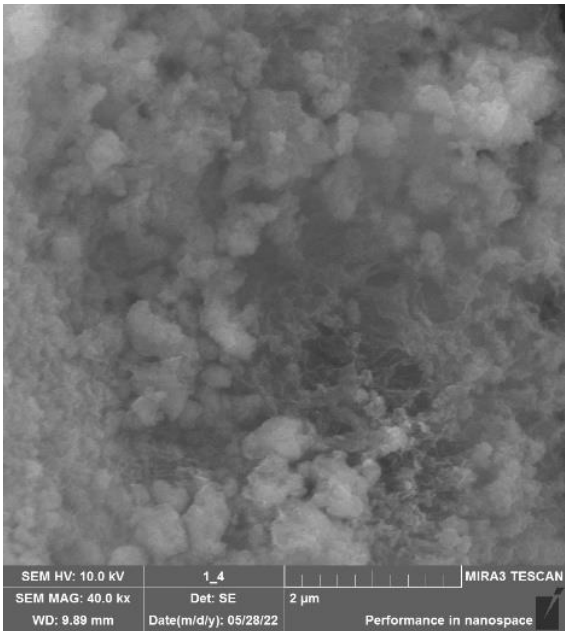
SEM HV: 10.0 kV
1_4
MIRA3 TESCAN
SEM MAG: 40.0 kx
Det: SE
2 µm
WD: 9.89 mm
Date(m/d/y): 05/28/22
Performance in nanospace

f

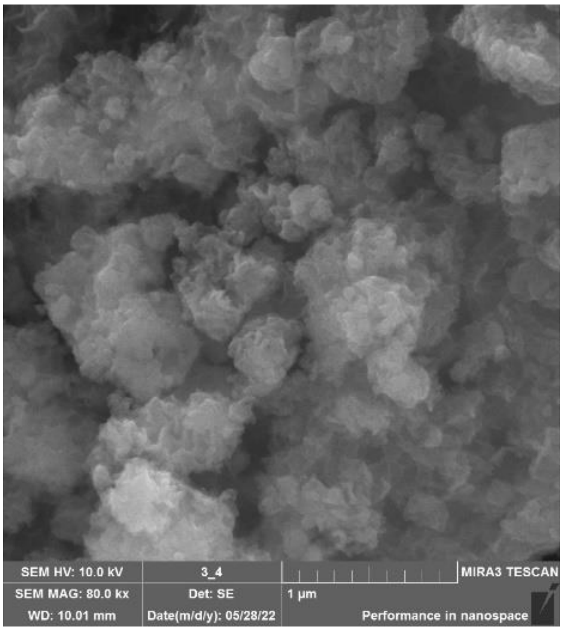
SEM HV: 10.0 kV
3_4
MIRA3 TESCAN
SEM MAG: 80.0 kx
Det: SE
1 µm
WD: 10.01 mm
Date(m/d/y): 05/28/22
Performance in nanospace

g

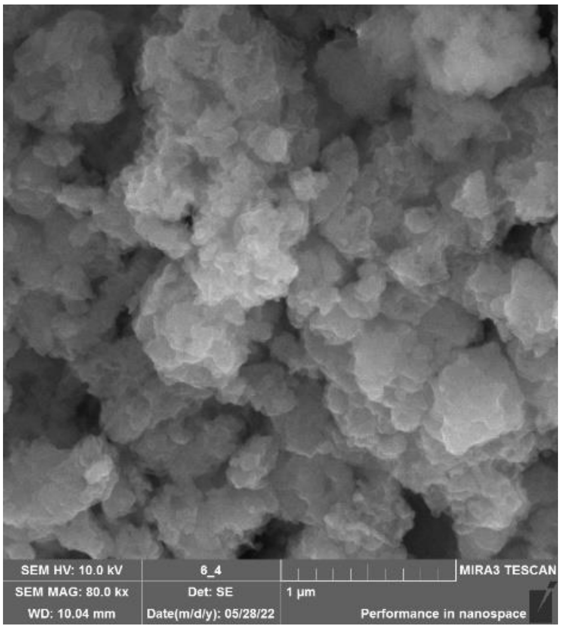
SEM HV: 10.0 kV
6_4
MIRA3 TESCAN
SEM MAG: 80.0 kx
Det: SE
1 µm
WD: 10.04 mm
Date(m/d/y): 05/28/22
Performance in nanospace

h

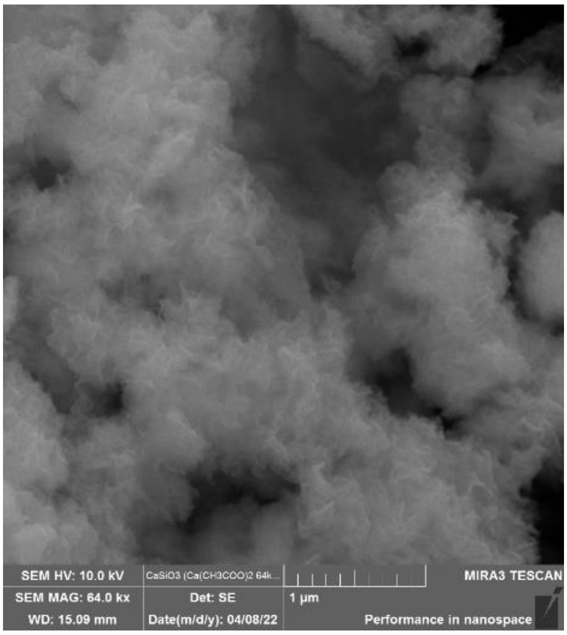

i

Figure S1. SEM micrographs of calcium silicate, stabilized with: a – L-valine, b – L-leucine, c – L-isoleucine, d – L-methionine, e – L-threonine, f – L-lysine, g – L-phenylalanine, h – L-tryptophan, i – without stabilizer.
